# Diabetes Cognitive Impairments and the Effect of Traditional Chinese Herbs

**DOI:** 10.1155/2013/649396

**Published:** 2013-12-09

**Authors:** Xiaohan Xu, Leilei Guo, Guoqing Tian

**Affiliations:** Department of Traditional Chinese Medicine, Peking Union Medical College Hospital, Peking Union Medical College, Chinese Academy of Medical Sciences, Beijing 100730, China

## Abstract

The problem of cognitive impairment resulting from diabetes is gaining more acceptance and attention. Both type 1 and type 2 diabetes mellitus have been proved to be associated with reduced performance on numerous domains of cognitive function. Although the exact mechanisms of cognitive impairments in diabetes have not been completely understood, hyperglycemia and insulin resistance seem to play significant roles. And other possible risk factors such as hypoglycemia, insulin deficiency, vascular risk factors, hyperactive HPA axis, depression, and altered neurotransmitters will also be examined. In the meanwhile, this review analyzed the role of the active ingredient of Chinese herbal medicine in the treatment of diabetes cognitive impairments.

## 1. Introduction

Diabetes mellitus (DM), characterised by chronic hyperglycemia, is one of the most important and prevalent chronic diseases [1]. The prevalence of total diabetes and prediabetes was 9.7% and 15.5% in adults (≥20 years old) in China, and diabetes is increasing with age (3.2%, 11.5%, and 20.4% among persons who were 20 to 39, 40 to 59, and ≥60 years of age, resp.) [2]. DM is a systemic disease that can damage any organ in the body, including both small and large vessels, central and peripheral nerves, retina, skin, and kidney. Although the term of diabetic encephalopathy has not been well established, significantly more interest has been dedicated to cognitive impairments in diabetes in recent years [3–6]. In addition, there are studies indicating that the risk of Alzheimer's disease (AD) (relative risk RR 1.4–2.4) and vascular dementia (VD) (RR 2.2–4.2) is increased in DM [7], especially in type 2 diabetes (T2DM) [8].

Diabetes is associated with various risk factors that may influence cognitive functioning, including diabetes-specific factors (e.g., hyperglycemia, insulin deficiency, and insulin resistance), vascular risk factors that are linked to diabetes (e.g., hypertension, hyperlipidemia, and obesity), and complications of (micro)vascular disease (e.g., stroke). Although the mechanisms underlying the development of cognitive dysfunction in diabetes have not been completely elucidated, hypotheses such as oxidative stress and insulin/insulin receptor-mediated signal transduction are gaining more attention. In this review, we will highlight the probable risk factors and the possibly involved mechanisms relating cognitive dysfunction in diabetes.

## 2. Cognitive Impairments and Diabetes Mellitus

### 2.1. Hyperglycemia

#### 2.1.1. The Effect of Hyperglycemia

It is well established that hyperglycemia, a shared characteristic in both type 1 diabetes mellitus (T1DM) and T2DM, plays an important role in cognitive decline in diabetes. There is a lot of cross-sectional and longitudinal studies that have shown that there is a close relationship between high blood glucose and cognitive decrements or dementia. A cross-sectional study related to young children with T1DM diagnosed in preschool age found similar performance between the diabetic and the healthy subjects in cognitive tests, but poor glycemic control with higher glycosylated hemoglobin (HbA1c) was related to lower general cognitive abilities, slower fine motor speed, and lower receptive language scores, indicating that young children with T1DM already demonstrate some negative cognitive effects in association with chronic hyperglycemia [9]. Streptozotocin- (STZ-) treated experimental type 1 diabetic rats, with chronic hyperglycemia, express cognitive impairments and abnormalities in hippocampal long-term potentiation (LTP), an activity-dependent form of synaptic plasticity, which is believed to be related to the cellular mechanisms of learning and memory [10].

Similar evidence exists from cross-sectional studies in patients with type 2 diabetes. As a research found that T2DM was associated with diminished cognitive function in each cognitive domain except for memory functions and the cognitive decline was significantly associated with HbA1c and duration of DM [11]. And other research results have confirmed that cognitive decrements in patients with type 2 diabetes were associated with longer diabetes duration [12] and elevated HbA1c levels [13]. Longitudinal studies in patients with type 2 diabetes also showed that longer diabetes duration and higher HbA1c levels were associated with a faster rate of cognitive decline [14–16]. Hyperglycemia may also lead to acute changes in cognitive functioning. Both type 1 and 2 diabetic patients were found to have an acute slower performance on the psychomotor task and mental subtraction speed and increased subtraction errors when blood glucose levels increased above 15 mmol/L [17]. Meanwhile, uncontrolled diabetes (blood glucose ≥11.0 mmol/L) [18] and diabetes duration [19] increase the risk of AD and VD.

#### 2.1.2. The Possible Mechanisms of Hyperglycemia Induced Cognitive Impairments

In view of the effect of chronic hyperglycemia on brain function, structure and micrangium, it may be not too much to hypothesize that hyperglycemia is a determinant of cognitive decline in patients with diabetes [20]. Although hyperglycemia may not lead to dementia directly, it could certainly reduce the threshold for dementia. However, the mechanisms through which hyperglycemia might mediate this effect are less clear, which we will discuss as follows.

Oxidative stress has been implicated as a contributor to both the onset and the progression of diabetes and its associated complications. Hyperglycemia can cause an increase in generation of reactive oxygen species (ROS), reactive nitrogen species (RNS), and oxidative stress markers, such as lipid peroxidation and protein oxidation, with an accompanying decrease in antioxidant levels, which have been suggested to be responsible for the induction of cognitive deficits often observed in diabetic rats [21, 22]. And these oxidative stress and cognitive impairments can be corrected in diabetic rats by the administration of antioxidants. Reduced glutathione levels, a critical intracellular antioxidant, and enzymatic activities of superoxide dismutase, an important enzymatic antioxidant, can be observed in both cerebral cortex and hippocampal regions of STZ-induced diabetic rats, accompanying with cognition deficits [23].

Hyperglycemia can also increase polyol pathway flux, enhancing the transformation of intracellular glucose into sorbitol via aldose reductase. Sorbitol cannot outflow the cell freely because of strong polarity, leading to the accumulation of sorbitol in cell [24]. Evidence showed that in STZ-induced diabetic rats, there is an increase in sorbitol in brain accompanied by a significant decline in long-term spatial memory compared with euglycemia rats. This accumulation of sorbitol was reduced significantly when the diabetic rats were treated with the aldose reductase inhibitor sorbinil, and long-term spatial memory was restored [25].

In addition, hyperglycemia can contribute to the increased production of advanced glycated end products (AGEs) [26]. The cell surface receptor of AGEs (RAGE) can lead to the production of intracellular ROS [27]. Diabetic mice with demonstrated cognitive impairments have been found to have increased expression of RAGEs in neurons in brain, suggesting a possible role of RAGEs in the development of cerebral dysfunction [28].

Finally, hyperglycemia can lead to the activation of the diacylglycerol (DAG) protein kinase C (PKC) pathway [29]. The PKC family consists of a number of different PKC isoforms, most of which are activated by the lipid second messenger DAG, which is oversynthesized under hyperglycemic conditions. Activated PKC contributes directly to the oxidative stress environment by activating reduced nicotinamide adenine dinucleotide phosphate (NADPH) oxidases, resulting in excessive ROS production. In alloxan diabetic untreated rats, expression of protein kinase C-*α* was shown to be significantly increased in brain, compared with the treated diabetic rat and control rats [30].

However, the above mechanisms are not independent of each other. Oxidative stress may trigger a cascade of events leading to neuronal damage and ultimately affect the cognition in diabetes. For example, in STZ-induced diabetic rats, levels of prooxidant compounds were all increased in the hippocampus, including RAGEs, galectin-3 (a proatherogenic molecule), and aldose reductase (the key enzyme in polyol pathway); whereas activity of the glycolytic enzyme glyceraedehyde-3-phosphate dehydrogenase (an antioxidase) was decreased, indicating elevated superoxide levels. When antioxidant, dehydroepiandrosterone, was administered to diabetic rats, this oxidative imbalance was improved significantly, reinforcing that oxidative stress plays a role in diabetes-related neuronal damage [31].

Otherwise, benefits of aggressive glucose management in type 2 diabetes are less clear. The Memory in Diabetes (MIND) substudy of the Action to Control Cardiovascular Risk in Diabetes (ACCORD) trial showed no benefit of intensive glucose-lowering therapy on cognitive function or total brain volume in a large population of people with type 2 diabetes during 40-month follow-up [32]. Similarly, ACCORD, ADVANCE (Action in Diabetes, and Vascular Disease: Preterax and Diamicron Modified Release Controlled Evaluation), and the Veterans' Affairs diabetes trial reported that intensive glucose control had no significant effect on macrovascular disease in people with type 2 diabetes [33–35].

### 2.2. Hypoglycemia

Hypoglycemia is a well-known complication of glucose-lowering therapy and severe hypoglycemic episodes may have detrimental effects on the brain. It is well established that an extended episode of profound hypoglycemia, such as one with a blood glucose level below 1.0 mmoL per liter, can induce massive cerebral energy failure with a corresponding development of neuronal necrosis [36]. Less severe episodes of hypoglycemia are also known to disrupt brain activity transiently, which can lead to short-term cognitive impairments, including difficulty in concentrating and speech, drowsiness, odd behavior, and incoordination [37]. A retrospective survey involving 16,667 type 2 diabetic patients with a mean age of 65 years showed a dose-response relationship between the occurrence of severe hypoglycemic episodes and the risk of developing dementia [38]. Childhood-onset type 1 diabetic patients who had experienced severe hypoglycemia demonstrate impaired declarative memory and spared nondeclarative memory functioning [39].

In contrast, The Diabetes Control and Complications Trial/Epidemiology of Diabetes Interventions and Complications (DCCT/EDIC) did not find evidence for an association between the frequency of severe hypoglycemic episodes and declined cognition in young adult patients (mean age 27 years) with type 1 diabetes [40]. Similarly, data from a systematic meta-analysis did not show any relation between recurrent hypoglycemia and performance in cognitive tests [41]. And the Fremantle diabetes study found no evidence that severe hypoglycemia contributes to cognitive decline in older patients (aged ≥70 years) with type 2 diabetes over the subsequent 5 years [42].

It seems that patients who are exposed to chronic or recurrent hypoglycemia may become remarkably tolerant to the state, so that the long-term effects of repeated hypoglycemic episodes on cognition are controversial [43]. In addition, the contradictory study results are possibly partly due to the heterogeneity of the samples and neuropsychological measures and the differential vulnerability of the brain to hypoglycemia in young and older patient populations [44]. Overall, a growing body of research suggests that moderate-to-severe hypoglycemia does not seem to result in substantial cognitive dysfunction, although that might not be true for some high-risk groups such as children diagnosed within the first few years of life [45, 46]. It seems that children with type 1 diabetes mellitus and developing brain may be more vulnerable to the adverse effects of hypoglycemia, while, in young adults, hypoglycemic events seem not easy to cause cognitive decrements by themselves, but, in view of the above results, hypoglycemia is not entirely benign; as for older individuals, the relationship between dementia or cognitive deficits and hypoglycemic events appears to be bidirectional: hypoglycemia predisposes to dementia and dementia predisposes to further hypoglycemic events.

### 2.3. Insulin Deficiency and Insulin Resistance

The absolute deficiency of insulin and insulin resistance are the basic pathophysiologic changes of type 1 and type 2 diabetes, respectively. The role of insulin deficiency and insulin resistance in the development of cognitive impairment in diabetes is gaining an increasing concern.

Inverse to traditional ideas, insulin crosses the blood-brain barrier and might even be produced locally in the brain [47]. Insulin is distributed throughout the brain, being abundant in the hippocampus, hypothalamus, and the cortex. Besides being a modulator of food intake and energy homoeostasis, insulin is also an important neurotrophic factor [48]. Interruption of insulin production and insulin receptor activity has been shown to cause deficits in learning and memory formation [49]. As clinical evidence, AD patients were found to have lower than normal cerebrospinal fluid (CSF) levels of insulin, whereas administration of insulin through intravenous infusion while keeping plasma glucose at a fasting baseline level significantly improves the cognitive performance of these patients [50].

Insulin is an important neurotrophic factor, nevertheless, hyperinsulinism is not good news for diabetic patients. Furthermore, there is no doubt that insulin is neurotrophic in moderate concentration, but the whole circumstances become more complicated in conditions of hyperinsulinism in diabetes. There is increasing evidence linking insulin resistance to cognitive decline and dementia in diabetes [51, 52].

For one thing, insulin resistance, resulting in compensatory hyperinsulinemia in type 2 diabetes, is associated with the abnormity of insulin/insulin receptor-mediated signal transduction. One of the key enzymes is glycogen synthase kinase 3 (GSK3), which is inactivated by phosphorylation in insulin/insulin receptor-mediated signal transduction, and generally opposes the actions of insulin. The impaired insulin/insulin receptor-mediated signaling pathway lead to activation of GSK3 by dephosphorylated, which is associated with the hyperphosphorylation of tau and increased production of amyloid-*β*-peptide (A*β*) [53, 54]. A*β* and tau protein, which are the main components of senile plaques and neurofibrillary tangles (NFTs), respectively, are the pathological hallmark of AD.

For another, too much insulin in the brain may be associated with reduced A*β* clearance due to competition for common principal scavenger, insulin degrading enzyme (IDE) [55]. IDE is a metalloprotease enzyme, responsible for insulin degradation and is also the main enzyme responsible for A*β* degradation. Since the affinity for the binding of insulin to IDE is much greater than the one for the A*β* peptide, hyperinsulinemia may lead to reduced A*β* peptide depurative mechanisms and accumulated concentration in the brain [56].

To sum up, deficiency of insulin secretion, the characterization of type 1 diabetes, may lead to the declined nutrition of neurons in the brain and eventually cause neuronal atrophy, apoptosis, and necrosis and cognitive decline. However, too much complement of exogenous insulin increases the risk of hypoglycemia, which is dangerous to cognitive function. Insulin resistance in type 2 diabetes can result in high phosphorylation of tau protein and the accumulation of A*β* peptide in brain indirectly via IDE or via insulin/insulin receptor signal pathway, leading to AD-like pathology and cognitive impairment. Actively improved insulin sensitivity may be favorable not only for blood glucose decreasing but also for cognitive improvement.

### 2.4. Vascular Risk Factors

Diabetes is often accompanied by vascular risk factors, such as hypertension, hypercholesterolemia, and obesity, which might also affect the brain and cognition. The Framingham study has shown that hypertension was a significant risk factor for poor cognitive performance in T1DM patients during the followed 28–30 years [57]. Another longitudinal study found that cognitive decrement in patients with type 2 diabetes was related to long-term exposure to hypertension, even in prediabetes stages [58]. Additionally, the highly variable extent of cognitive dysfunction seen in adults with type 2 diabetes might be caused by the presence of several comorbid disorders, such as hypertension and obesity. Indeed, a study of elderly people with type 2 diabetes showed that their learning and memory skills were significantly disrupted, but when the analysis was adjusted for the presence of hypertension this difference was not significant [11]. Dyslipidemia and obesity also seem associated with cognitive functioning in patients with diabetes. As there is evidence showing that obesity was associated with decreased verbal declarative memory performance in middle-aged patients with diabetes [59]. And longitudinal study in old patients with diabetes found that higher baseline total cholesterol level and higher baseline waist-to-hip ratio were associated with a decreased risk of cognitive impairment [19]. And the use of lipid-lowering drugs was associated with less severe brain magnetic resonance imaging (MRI) abnormalities in T2DM patients [60].

However, not all the studies have the coincident results. In fact, there are studies that actually reported that high blood pressure and high cholesterol were associated with a decreased risk of dementia [61, 62]. One research found that hypertension and hypercholesterolemia increased the risk of dementia in diabetes, particularly, if these risk factors were present at midlife; in older age groups, however, the relation between vascular risk factors and cognitive decline was generally less consistent [63]. This may explain why longitudinal studies in older (>70 years) patients with diabetes report inconsistent results on the relation between vascular risk factors and cognitive impairment or dementia. Maybe the old patients need higher blood pressure levels to maintain an adequate cerebral perfusion, which still needs verification. In addition, most studies concerning diabetes and cognition have adjusted the vascular risk factors; it seems that hypertension, hypercholesterolemia, or obesity are not the only determinants that contribute to cognitive decline or dementia in diabetes.

However, how these risk factors may exert their influence is not well understood. But it is well established that the vascular risk factors of diabetes are enhancing the risk of vascular diseases, decreasing cerebral blood flow, and activating inflammation, which may be responsible for the development and progression of cognitive disorders in diabetes.

Vascular risk factors are typically thought to produce deleterious effects on brain function via overt strokes. Patients with diabetes have a 2- to 6-fold increased risk in stroke [64]. And diabetes is also one of the most consistent predictors for recurrent stroke, even independent of glucose control [65]. Stroke has long been hypothesized to contribute to abnormalities in cognition. In addition, patients with diabetes have also been found to have decreased global rates of cerebral blood flow [66] and thickened brain capillary basement membranes [67], which may exert their contribution in cognition deterioration in diabetes. Finally, studies have demonstrated the existence of a chronic systemic inflammatory state in diabetes, including endothelial dysfunction, platelet hyperactivity, increased inflammatory markers such as C-reactive protein, interleukin-6 (IL-6), and tumor necrosis factor-*α* (TNF-*α*) [68–70].

In conclusion, the majority of cross-sectional and longitudinal studies have confirmed the association between vascular risk factors and cognitive decrements in diabetes. However, according to the complex circumstances in diabetes with vascular risk factors, it is not sure about how these factors contribute their role to the development of cognitive impairment.

### 2.5. HPA Axis Dysfunction

The hypothalamic-pituitary-adrenal (HPA) axis is a complex set of interactions between the hypothalamus, the pituitary gland, and the adrenal or suprarenal glands. While exposure to acute stress facilitates memory formation and consolidation, chronic stress or chronic exposure to stress levels of glucocorticoids impairs cognitive performance. Impairment in hypothalamic-pituitary-adrenal (HPA) axis function is proposed to participate in the experimental models of type 1 and type 2 diabetes, as well as diabetic subjects in poor glycemic control [71]. Although it is not known whether glucocorticoids are involved in cognitive dysfunction in diabetes in human, studies of rodents have provided evidence that elevated adrenal glucocorticoids or impairment in HPA axis can be found in diabetic rats with cognitive function deficits [72].

Firstly, there is evidence that chronic overexposure to corticosterone, one kind of the glucocorticoids, can lead to impaired cognition [73]. In diabetic rats with cognition deficits, the HPA axis is chronically stimulated, and the administration of glucocorticoid antagonist can attenuate the cognitive deficits of diabetic rats [74].

In addition, high corticosterone can impair synaptic plasticity and cause deficits in cognitive function [75, 76]. There are studies which found increased glucocorticoid secretion, mild cognitive deficits in diabetic rodents, as well as deficits in neurogenesis and synaptic plasticity of hippocampus, which can all be reversed when normal physiological levels of corticosterone are maintained [72, 77].

Finally, there are several studies in human, which are with poorly controlled diabetes and cognition decline, show hyperactivation of the HPA axis and elevated circulating cortisol [78, 79]. And the blocking of circulating inert cortisone to regenerate active glucocorticoid cortisol seems to improve verbal fluency and verbal memory in aging diabetic patients [80]. As both endogenous and exogenous increase of glucocorticoids could induce cognitive impairments [81].

It is therefore possible that HPA axis dysfunction and/or exposure to elevated glucocorticoid levels may contribute to the cognitive deficits in diabetes. Although the cause and effect have not been determined in many of these situations, these findings may place impairment in HPA axis function at the important area of diabetes-induced cognitive impairment.

### 2.6. Depression

Researches show that patients with diabetes double the odds of comorbid depression compared to nondiabetic persons [82, 83], which may negatively affect cognitive performance and daily activities in diabetic patients. And the relation between diabetes and depression seems to be bidirectionally, as depression may also increase the risk of developing diabetes mellitus [84]. Longitudinal analysis reported that a high level of depressive symptomatology is not predictive of cognitive deterioration, but cognitive deterioration was associated with the concomitant level of depressive symptomatology cross-sectionally [85].

The exact nature of the relation between diabetes and depression is unclear. There is study that suggests that depression in diabetes may disturb the balance of brain-derived neurotrophic factor (BDNF), causing cognitive decline or dementia [86]. Another study found that cerebrovascular risk factors were significant predictors of depression in diabetic patients [87], suggesting that depression-related cognitive deficits may be the result of cerebrovascular diseases.

Although depressive symptoms may affect cognitive performance, the majority of studies assessed cognition in relation to diabetes controlled for the potential confounding effects of depression. It can be concluded that depression is not the sole risk factor in diabetes-associated cognitive decrements.

### 2.7. Neurotransmitter

Altered neurotransmitter function has been observed in diabetic rodent models and may also contribute to cognitive dysfunction. Correlations between lifetime glycemic control and increased frontal brain glucose and neurotransmitter concentrations measured with proton magnetic resonance spectroscopy in adults with type 1 diabetes do suggest an underlying pathophysiological change [88]. People older than 60 years with type 2 diabetes do not show abnormalities in other neurotransmitters and metabolites, whereas younger adults with type 1 diabetes show abnormalities in concentrations of myoinositol, choline, and N-acetylaspartate (a marker of neuronal damage) [89].

Cholinergic neurotransmission is a crucial process underlying memory and cognitive function. One of the most important mechanisms responsible for correct cholinergic function is performed by enzyme choline esterase (ChE). Several studies have found an increased ChE activity in brain and consequently can be associated with cognitive impairments in diabetic rats [90, 91]. On the meanwhile, it was reported that the activity of acetylcholinesterase (AChE, a neurotransmitter associated with learning and memory) was increased in serum and cerebral cortex of diabetic rats [92], which may contribute to cognitive deficits exhibited in diabetes. And the increased levels of 5-hydroxytryptamine (5-HT) has been demonstrated in diabetic rat brain and can be repaired by insulin administration [30]. In STZ-induced diabetic rats, N-methyl-d-aspartate (NMDA) receptor-dependent and NMDA receptor-independent LTP were impaired in hippocampus, contributing to learning and memory deficits [93]. Alterations in glutamate turnover may contribute to diabetes mediated deficits in hippocampal synaptic plasticity. As glutamate receptors were upregulated in the brain of diabetic rats, accompanied by impairment in the expression of hippocampal LTP. And this phenomenon was prevented by insulin treatment [94].

## 3. The Therapeutic Function of the Active Ingredient of Traditional Chinese Herbs in Diabetes Cognitive Impairments

Traditional Chinese Herbs are becoming the important sources for the development of antidiabetic drugs. A wealth of studies have described the effects of constituents from herbs or plants on diabetes-associated cognitive deficits in rodents by several aspect. There are more and more reports concerning the therapeutic function of the effective component of traditional Chinese herbs in diabetes cognitive impairments.

### 3.1. Attenuating AGEs Mediated Neuroinflammation

Danshensu, a compound from *Salvia miltiorrhiza* Bunge, is widely used in China, Korea, Japan, and other Asian countries in the treatment of cerebrovascular diseases. C57BL/6 mice with streptozotocin injected intraperitoneally were administered with sodium Danshensu by gavage once a day for 12 weeks. The western blot analysis revealed that Danshensu partly blocked the expression of RAGE, p-p38, and COX-2 and NF-*κ*B activation and inhibited the increase of TNF-*α*, IL-6, and prostaglandin E_2_ (PGE_2_) in the hippocampus tissues of the diabetic mice [95]. In the meantime, Danshensu not only reduced the mean escape latency but also increased the percentage of time spent in the target quadrant in the Morris water maze. However, Danshensu did not affect body weight and the levels of blood glucose, glycosylated hemoglobin, and insulin [95]. This study showed that treatment with Danshensu had effect on learning and memory deficits in diabetic mice which was not associated with the ability of Danshensu to regulate blood glucose. These findings demonstrate that Danshensu may provide a potential alternative for the prevention of cognitive impairment associated with diabetes by attenuating AGE-mediated neuroinflammation.

### 3.2. Reducing the Accumulation of A*β* in Brain

Rhizoma Anemarrhenae, the dried rhizome of *Anemarrhena asphodeloides* Bunge, is a well-known Chinese Materia Medica. The learning ability of the diabetic rats treated with total saponins from Rhizoma Anemarrhenae was significantly augmented in the Morris water maze test compared with the other diabetic rats. And the experimental rats showed both reduced A*β*(1–40) and A*β*(1–42) levels in brain. In the meanwhile, Rhizoma Anemarrhenae decreased the blood glucose level and increased the body weight of the diabetic rats [96]. This research demonstrated that Rhizoma Anemarrhenae had a markedly inhibitory effect on the elevations of A*β*(1–40), A*β*(1–42), and the blood glucose levels of STZ-induced diabetic rats, which maybe was one of the reasons for the prophylactic efficacy of Rhizoma Anemarrhenae to diabetes-associated cognitive decline.

### 3.3. Anti-Inflammation and Antioxidation

A growing body of literatures suggests that oxidative stress and inflammation are involved in the pathogenesis of late diabetes complications. Hyperglycemia reduces antioxidant levels and concomitantly increases the production of free radicals. Hyperglycemia is also associated with the enhanced inflammatory response [97, 98]. Evidences demonstrate that inflammation is involved in the pathogenesis of diabetes-associated cognitive decline [23].

Ginsenoside Re (Re), a triterpenoid saponin compound derived from *Panax ginseng* C. A. Mey., shows a remarkable antidiabetic effect. Chronic Re treatment decreased escape latency and increased percentage of time spent in the target quadrant of the diabetic animals. Furthermore, Re treatment reduced the levels of TNF-*α* and malondialdehyde (MDA, an important marker for lipid peroxidation) in brain areas, as well as enhanced GSH levels in serum of diabetic rats, compared to the untreated diabetic rats. Meanwhile, chronic Re treatment markedly decreases the fasting blood glucose level of diabetic rats [99]. In this study, learning and memory abilities are significantly impaired in STZ-induced diabetic rats, accompanied by the markedly elevated oxidative stress and inflammation. But chronic administration of Re could remarkably attenuate cognitive deficits caused by diabetes and could significantly decrease oxidative stress and inflammation of diabetic rats, suggesting that, besides decrease blood glucose, anti-inflammation and antioxidation may contribute to the amelioration of diabetes-associated cognitive decline.

Berberine is an isoquinoline alkaloid, which is mainly found in *Coptis chinensis*. Chronic treatment with berberine improved cognitive performance, lowered hyperglycemia, decreased lipid peroxidation, and increased the reduced glutathione of the diabetic animals [100]. Therefore, the restoration of cognitive function observed in diabetic rats in this study may be due to the ability of berberine to reduce hyperglycemia and oxidative stress. Chronic lycopene (a carotenoid mostly found in tomatoes and tomato products) treatment had the effect of anti-inflammation and antioxidation and significantly improved the blood glucose levels and the cognitive function of diabetic rats [90]. Chronic treatment with curcumin (a polyphenolic flavonoid found in turmeric and several traditional herbal medicine) improved the blood glucose levels and attenuated cognitive deficit, oxidative stress, and inflammation in diabetic rats [101]. Chronic supplementation with the extracts of *Withania somnifera* and Aloe vera lowered the blood glucose level, reduced the oxidative damage, and improved memory impairment and motor dysfunction of diabetic rats [102].

### 3.4. Restoring Hippocampal Synaptic Plasticity and Antiapoptotic

Diabetes gradually impairs hippocampus-dependent cognitive functions and synaptic plasticity [103, 104]. Part of deficits in performing the spatial learning and memory tasks in diabetes is attributed to induction of apoptosis of CA1 pyramidal neurons [105]. Chronic berberine treatment reduced serum glucose level, ameliorated learning and memory impairment, restored population spike amplitude and field excitatory postsynaptic potential, and attenuated apoptosis of pyramidal neurons in the CA1 area of STZ-diabetic rats [106]. Since berberine could restore hippocampal synaptic plasticity and inhibit apoptosis, this may have attenuated learning and memory abnormalities in diabetic rats.

### 3.5. Protecting the Cholinergic Function

Cholinergic neurotransmission is a crucial process underlying memory and cognitive function. Chronic treatment with lycopene could prevent the rise of AChE activity in the cerebral cortex of STZ-treated diabetic rats [90]. Berberine could decrease the ChE activity and ameliorate cognitive deficits in diabetic rats [100]. Chronic treatment with curcumin significantly ameliorated the diabetes cognitive deficits and cholinergic dysfunction [101].

The beneficial effects of the active ingredient of traditional Chinese herbs observed in all these studies above could be multivariate. The active ingredient of Traditional Chinese herbs could ameliorate the diabetes cognitive dysfunction by attenuating AGEs mediated neuroinflammation, reducing the accumulation of A*β* in brain, anti-inflammation, antioxidation, restoring hippocampal synaptic plasticity, antiapoptotic, and protecting the cholinergic function. Most of the active ingredients of Traditional Chinese herbs could improve the blood glucose levels except Danshensu, which did not affect the levels of blood glucose of the diabetic animals. Since that chronic treatment with Danshensu had not regulate blood glucose in diabetic mice, partial restoration of cognitive functions observed in diabetic animals in that research may not be associated with the ability of attenuating hyperglycemia. At present the multifactorial pathogenesis of cognitive and memory impairments in diabetes has not yet completely been understood. Whether the other Traditional Chinese Herbs which have the inhibitory effect on the elevations of the blood glucose levels directly improved diabetes-induced cognitive impairments or indirectly through the correction of hyperglycemia have not been identified.

## 4. Summary and Future Directions

In conclusion, there have been significant gains in our understanding of the effect of diabetes on cognitive impairment. As substantial researches suggest that diabetes increases the risk of mild cognitive impairment and hastens the progression to dementia, cognitive impairment should be listed as one of the chronic complications of diabetes. Lots of risk factors such as hyperglycemia, hypoglycemia, insulin deficiency, insulin resistance, vascular risk factors, HPA axis dysfunction, depression, and altered neurotransmitters have been proved to be associated with cognitive decline in diabetes. It is unlikely that any single factor is solely responsible for the development of cognitive deficits in diabetic subjects. Rather, these factors may act in additive or synergistic ways to impair neuronal homeostasis, increase neuronal vulnerability, and eventually contribute to cognitive decline. The pathogenesis of cognitive dysfunction in diabetes is only partially understood. Chronic hyperglycemia-mediated oxidative stress and insulin resistance associated abnormity in insulin/insulin receptor signal pathway are the focus in the current researches.

Although much research has been done on the influence of diabetes mellitus on cognitive function, still many questions remain. Firstly, it is not clear whether cognitive impairments seen in cognitive testing result in meaningful deficits in the daily life of the patients as studies show relatively light to mild decrements in cognitive functioning, which slowly progress over time. Given the subjective nature of assessing livelihood activities, it may be difficult to address this question. However, it can be inferred that the impact of cognitive impairment on diabetic patients will become more outstanding following the enhanced average life span, increased requirements for life quality, and raising prevalence of both diabetes and dementia around the world. Hence another key target for future studies is to find ways to prevent diabetes-associated cognitive dysfunction, particularly for dementia. Now that clinical and experimental researches have found some important risk factors in cognitive decline in diabetes; further researches need to verify if improving these risk factors, for example, improving insulin sensitivity, can decrease or prevent the occurrence of cognitive decline and dementia. Surprisingly, diabetes might not increase the rate of cognitive decline over time. When people aged 56–80 with diabetes were followed up for 4 years, their overall cognitive decline, measured by a comprehensive battery of cognitive tests, did not differ significantly from that of age-matched participants without diabetes [107]; it shows that diabetes-related cognitive changes develop slowly over a prolonged period of time, probably much longer than 4 years.

Moreover, we should gain more insight into the etiology and mechanisms that drive accelerated cognitive decline in diabetes, which is important in treatment of dementia or cognitive decline in diabetes. Although oxidative stress is gaining acceptance as the key mechanism of peripheral neuropathy of diabetes, whether it is the same situation in central neuropathy complication of diabetes is unascertained. And as the association between AD and insulin resistance syndrome is gaining attention [108], insulin/insulin receptor-mediated signal transduction in diabetes is also worth further research. At present, the studies about the therapeutic function of Traditional Chinese medicine in diabetes cognitive dysfunction remain based on the monomer of herbal medicine. The compound of traditional Chinese medicine consisting of several medicinal materials which containing numerous ingredients has varied biological actions. The researches about the compound of traditional Chinese medicine on the treatment of this disease is worthy of in-depth study.
